# Combined In Vitro and Computational Investigations on Synthesized Sulfonamide-Based Antidiabetic Agents

**DOI:** 10.3390/ph19040538

**Published:** 2026-03-26

**Authors:** Ancuța Dinu Iacob, Oana Cioancă, Iuliana Aprodu, Rodica Tatia, Andreea-Teodora Iacob, Cornelia Mircea, Dana Tutunaru, Alexandra Burlacu Pavel, Ionut-Iulian Lungu, Oana Maria Dragostin

**Affiliations:** 1 Research Centre in the Medical-Pharmaceutical Field, Faculty of Medicine and Pharmacy, “Dunărea de Jos” University of Galati, 800201 Galati, Romania; ancuta.dinu@ugal.ro (A.D.I.); dana.tutunaru@ugal.ro (D.T.); alexandra.burlacu@ugal.ro (A.B.P.); 2 Grigore T. Popa University of Medicine and Pharmacy, 700115 Iasi, Romania; oana.cioanca@umfiasi.ro (O.C.); cornelia.mircea@umfiasi.ro (C.M.); ionut-iulian.lungu@umfiasi.ro (I.-I.L.); 3 Department of Food Science, Food Engineering, Biotechnology and Aquaculture, Faculty of Food, Science and Engineering, “Dunărea de Jos” University of Galati, 800201 Galați, Romania; iuliana.aprodu@ugal.ro; 4 Department of Cellular and Molecular Biology, National Institute of Research and Development for Biological Sciences, 060031 Bucharest, Romania; rodica.tatia@gmail.com; 5 Department of Pharmaceutical Sciences I, Faculty of Pharmacy, Grigore T. Popa University of Medicine and Pharmacy, 700115 Iasi, Romania

**Keywords:** diabetes mellitus, sulfonamide, α-amylase, α-glucosidase, isocyanate

## Abstract

**Background/Objectives:** Worldwide, diabetes is a 21st century disease with continuously increasing prevalence. Current medications often have long-term adverse effects, which is why new substances are needed to help combat these disadvantages. **Methods:** In this respect, the present study develops a series of compounds with potential antidiabetic activity, including synthesis, physicochemical–spectral characterization and in vitro–in silico evaluation. **Results**: The sulfonamide derivatives were obtained by condensation reactions of para-toluenesulfonamide (**p-TSA**) with two different isocyanates, directly or after the condensation reaction with urea. The spectroscopic methods, IR, ^1^H-NMR, ^13^C-NMR, were used for the structural elucidation of the compounds to confirm the presence of the functional groups responsible for the antihyperglycemic action, namely amide, azomethine and sulfonyl groups. Cytotoxicity screening on NCTC fibroblasts confirmed the excellent safety profile of the most synthesized derivatives across the tested range (100–1500 μg/mL). In contrast, the **p-TSA-c-d** derivative showed a clear transition from a biocompatible profile at 100 μg/mL to a more cytotoxic phenotype at concentrations exceeding 750–1500 μg/mL. The synthesized derivatives, particularly **p-TSA-c-d**, exhibited remarkable antidiabetic potential by effectively inhibiting α-amylase and α-glucosidase, with IC_50_ values as low as 46.54 μM, outperforming the standard reference acarbose. The molecular docking tests revealed different mechanisms for the inhibitory activity exerted by the **p-TSA** derivatives on the two targeted enzymes. **Conclusions**: Although these developed compounds can be considered promising antidiabetic agents, studies can be further deepened in the future by performing in vivo tests.

## 1. Introduction

In 2024, there were an estimated 589 million cases of diabetes worldwide among adults aged 20–79 years, with projections suggesting this figure will reach 853 million cases within the next 25 years [1]. Due to the complications that develop both at microvascular and macrovascular levels, the management of DM is becoming increasingly difficult [2,3]. Compared to DM type I (DMT I), DM type II (DMT II) is more prevalent, requiring diverse therapeutic approaches [4]. However, various studies suggest the essential role of oral antidiabetic drugs, such as those in the sulfonamide class [5,6,7].

Although newer generation drugs are available, sulfonamides continue to be a first-line pharmacotherapy for DMT II in many parts of the world due to their efficacy, low cost, and treatment adherence [8,9,10]. Historically, the first oral antidiabetics with a sulfonamide structure appeared after 1955 (such as carbetamide, tolbutamide, tolazamide, chlorpropamide, acetohexamide) [11,12]. Since then, synthetic oral antidiabetic drugs have evolved into various classes grouped according to origin, chemical structure and mechanism of action (such as meglitinides, biguanides, thiazolidinediones, α-glucosidase inhibitors, dipeptidyl peptidase 4 inhibitors, sodium–glucose co-transporter 2 inhibitors, cycloset) [13,14]. Considering their therapeutic advantages, the present study aims to create a new series of sulfonamide compounds to treat DMT II, using p-toluenesulfonamide (**p-TSA**) as initial compound [15]. Thus, this work aimed to create a series of antidiabetic sulfonamide compounds, using **p-TSA** as a parent compound.

Also known as 4-methyl-benzene-1-sulfonamide, according to the International Union of Pure and Applied Chemistry (IUPAC), **p-TSA** has gained interest among scientists due to its notable pharmacological properties, and is intensively studied for its antitumor and antibacterial activities [15,16]. The benefits of its use include low toxicity and also its stability in acidic, neutral and alkaline environments [17]. A study by Richter D. et al. highlighted the minimal toxicity of this substance, showing a low risk of environmental pollution and low toxicity to fish, while the novelty of this research lies in the antidiabetic activity evidenced by **p-TSA** derivatives obtained through chemical synthesis [18].

Some antidiabetic therapies aim to reduce postprandial hyperglycemia by inhibiting mainly two enzymes, α-amylase and α-glucosidase, which are involved in the initial stages of carbohydrate metabolization [19]. A variety of performing antidiabetic medications share a common structural feature: the sulfonylurea group [20,21]. Glycyclamide, which has been used for many years in the treatment of type 2 diabetes, is a sulfonylurea compound that contains a cyclohexyl ring, having the **p-TSA** residue in its structure. Researchers have recognized it as an important compound due to its effects on blood glucose levels, and it has been utilized in numerous studies aimed at developing new antidiabetic drugs or formulations. The presence of the cyclohexyl ring gives this compound unique and remarkable pharmacokinetic and pharmacodynamic properties, making it a reference compound for the new derivatives being investigated in this research [22].

Additionally, the incorporation of isocyanates and urea in the development of new compounds offers significant advantages due to its well-characterized chemical structure [23,24,25,26].

Starting from this idea, the aim of this study was to obtain **p-TSA** derivatives (through various condensation reactions with isocyanates) and to combine the in vitro and in silico methods for evaluating their biological activity.

## 2. Results

### 2.1. Synthetic Approach of Aryl-Sulfonamides Derivatives

**Scheme 1:**
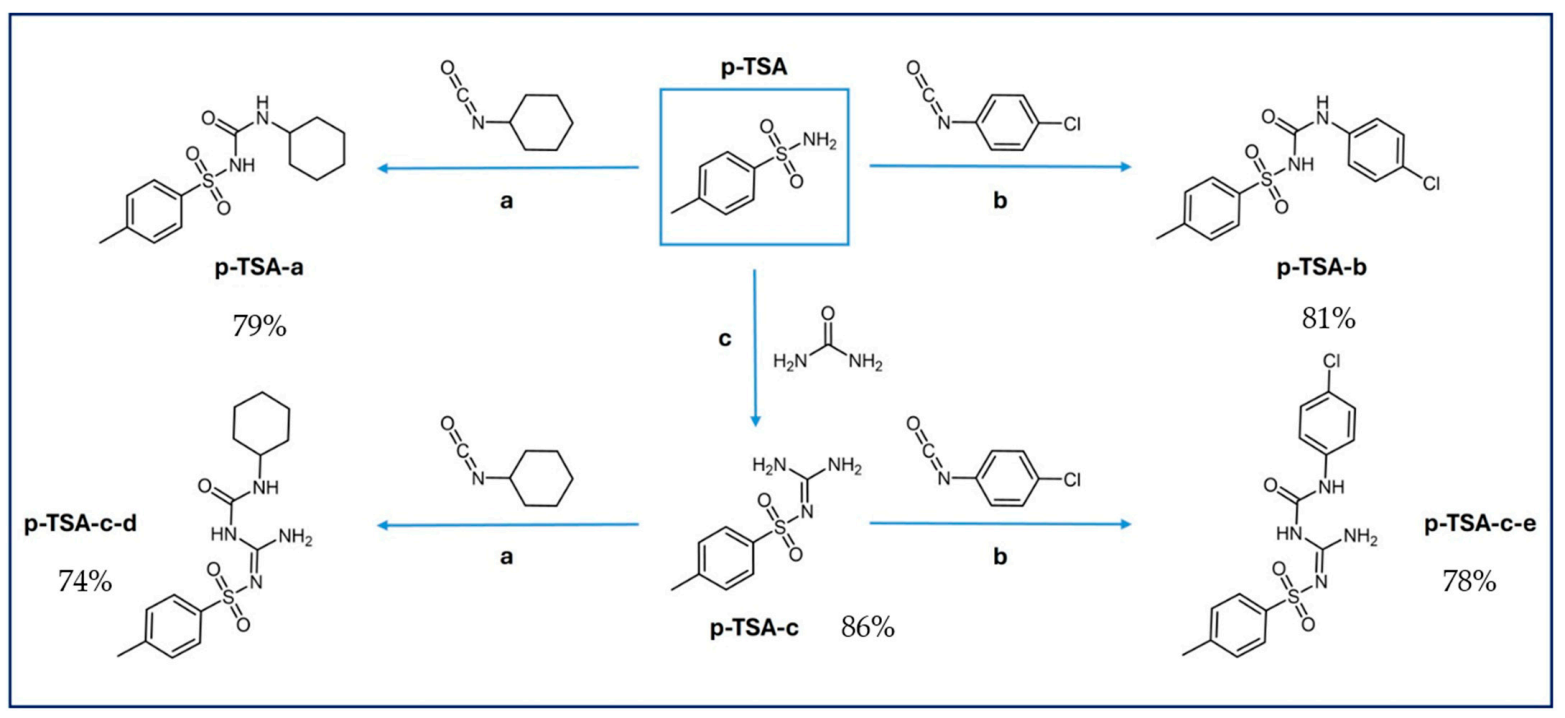
Synthetic approach for obtaining five sulfonamide compounds starting from **p-TSA** in the presence of K_2_CO_3_ as catalyst. Arrows indicate the reaction direction. Labels **a**, **b**, **c** represents the reagents used: (**a**) cyclohexyl isocyanate; (**b**) 4-chlorophenyl isocyanate; (**c**) urea. The percentages represent the yields of the synthesized compounds.

The selected synthetic approach for obtaining five new sulfonamide compounds, each starting from 4-methyl-benzene-1-sulfonamide (**p-TSA**), is shown in Scheme 1, while the compounds were thus obtained in yields ranging between 74% and 86%.

### 2.2. Analytical and Spectroscopic Characterization of the Synthetic Compounds

#### 2.2.1. Experimental Physicochemical Characterization

The organoleptic properties of each synthetic derivative were evaluated and are listed in Table 1. Visual control of the powders was carried out to identify the form in which they are present. The colors of the powders were determined by examining them spread on filter paper under natural light. The melting point was used as a straightforward and practical way to determine the purity of the synthesized compounds.

The new compounds were obtained as amorphous or crystalline powders, with a white color and having a specific odor. All the substances obtained showed one single melting point, which varied from 139 °C up to 185 °C, indicating their purity without intermediate or side products being present.

The solubility of the newly obtained compounds was tested by dissolving them in various solvents. The data are presented in Table 2.

#### 2.2.2. ATR-FTIR Spectroscopy

**Figure 1:**
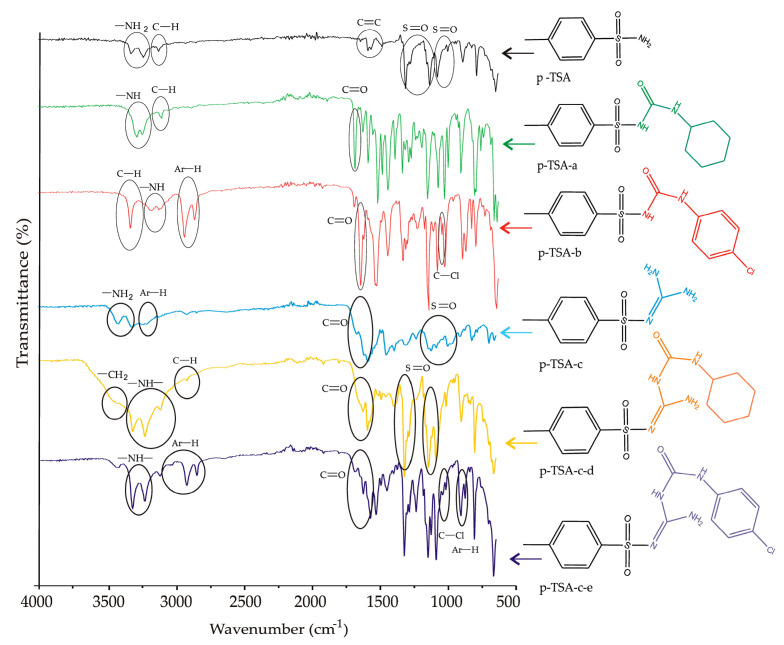
The IR spectrum of **p-TSA** (black line); **p-TSA-a** (green line); **p-TSA-b** (red line); **p-TSA-c** (blue line); **p-TSA-c-d** (yellow line); **p-TSA-c-e** (purple line).

The chemical structure of the compounds was confirmed by ATR-FTIR spectroscopy, where the characteristic absorption bands of all functional groups are indicated in the spectral region 4000–500 cm^−1^ (as shown in Figure 1).

The IR spectra provided definitive evidence for the formation of the target sulfonylurea derivatives. The most significant diagnostic marker was the appearance of a strong absorption band in the 1685–1710 cm^−1^ range, assigned to the carbonyl stretching vibration (C=O) of the newly formed urea linkage, a feature notably absent in the precursor **p-TSA**. Specifically, the cyclohexyl derivative (**p-TSA-a**) exhibited this band at 1705 cm^−1^, while the chlorinated analog (**p-TSA-b**) and the urea-condensed product (**p-TSA-c**) showed slightly shifted vibrations at 1685 cm^−1^ and 1680 cm^−1^, respectively. The structural integrity of the sulfonyl scaffold was confirmed by the persistent asymmetric and symmetric *SO*_2_ stretching doublets centered at 1340 cm^−1^ and 1160 cm^−1^. Furthermore, the secondary amide character of the N-substituted derivatives was supported by the simplification of the N–H region, whereas the terminal amino group in **p-TSA-c** was identified by its characteristic doublet at higher wavenumbers (3320–3410 cm^−1^).

The functionalization of the p-toluenesulfonylurea scaffold (**p-TSA-c**) with various isocyanates yielded two novel biuret derivatives with distinct physicochemical properties. The spectrum of **p-TSA-c** (blue line) displays a characteristic primary amine doublet in the 3320–3410 cm^−1^ region and a sharp carbonyl absorption (C=O) at 1680 cm^−1^, signaling the successful condensation with urea. Following the reaction with cyclohexyl isocyanate, the spectrum of **p-TSA-c-d** (yellow line) exhibits significant intensification of the aliphatic C-H stretching vibrations at 2932 cm^−1^ and 2856 cm^−1^, alongside a broadening of the carbonyl band, which reflects the presence of the dual C=O systems within the biuret scaffold. Furthermore, the synthesis of the chlorinated aromatic derivative (**p-TSA-c-e**, purple line) is confirmed by the emergence of additional aromatic C–H vibrations and a distinct diagnostic band at 1090 cm^−1^, corresponding to the C–Cl stretching. In both biuret derivatives, the disappearance of the sharp –NH_2_ doublet of the precursor in favor of broader secondary amide absorptions further validates the nucleophilic addition of the terminal amine to the respective isocyanates.

#### 2.2.3. NMR Analysis of the Synthesized Compounds

The chemical structure of the compounds obtained at the different stages of the anticipated reaction pathway was confirmed using ^1^H-NMR and ^13^C-NMR spectral analysis, by using DMSO-*d*_6_ as solvent.

*N–(cyclohexylcarbamoyl)–4–methylbenzenesulfonamide* (**p-TSA-a**) (Figure S1): ^1^H NMR (500 MHz): δ 1.28–1.70 (m, 10H, cyclohexyl ring: H_2_16, H_2_17, H_2_18, H_2_19, H_2_20), 2.32 (s, 3H, CH_3_), 3.87 (m, 1H, cyclohexyl ring: H_2_15), 6.54 (s, 1H, NH), 7.32 (d, 2H, H2, H4 orto vs. CH_3_), 7.52 (s, 1H, NHSO_2_), 7.71 (d, 2H, H1,H5 orto vs. SO_2_). ^13^C NMR: δ 21.4 (1C), 24.8 (2C), 25.7 (1C), 32.9 (2C), 48.6 (1C), 127.3 (2C), 129.7 (2C), 136.1 (1C), 144.9 (1C), 153.1 (1C).

*N–[(4–chlorophenyl)carbamoyl]–4–methylbenzene–1–sulfonamide* (**p-TSA-b**) (Figure S2): ^1^H NMR (500 MHz): δ 2.33 (s, 3H, CH_3_), 6.5 (s, 1H, NH), 7.32 (m, 4H, ArH: H2, H4 orto vs. CH_3_; H17, H19 orto vs. Cl), 7.52 (s, 1H, NHSO_2_), 7.71 (m, 4H, ArH: H1, H5 orto vs. SO_2_; H16, H20 orto vs. NH). ^13^C NMR: δ 21.4 (1C), 121.6 (2C), 127.0 (1C), 127.3 (2C), 129.0 (2C), 129.7 (2C), 130.8 (1C), 136.1 (1C), 136.2 (1C), 144.9 (1C).

*N–(diaminomethylidene)–4-methylbenzene–1–sulfonamide* (**p-TSA-c**) (Figure S3): ^1^H NMR (500 MHz): δ 2.31 (s, 3H, CH_3_), 7.29 (d, 4H, 2NH_2_), 7.30 (d, 2H, ArH: H2,H4 orto vs. CH_3_), 7.67 (d, 2H, ArH: H1, H5 orto vs. SO_2_). ^13^C NMR: δ 21.4 (1C), 127.1 (2C), 129.6 (2C), 136.8 (1C), 144.9 (1C), 159.3 (1C).

*N–{amino[(cyclohexylcarbamoyl)amino]methylidene}–4–methylbenzene–1–sulfonamide* (**p-TSA-c-d**) (Figure S4): ^1^H NMR (500 MHz): δ 1.36 (m, 6H, cyclohexyl ring: H20, H21, H22), 1.75 (m, 4H, cyclohexyl ring: H19, H23), 2.32 (s, 3H, CH_3_), 3.86 (d, 1H, cyclohexyl ring: H18), 6.5 (s, 2H, 2NH), 7.25 (s, 2H, NH_2_), 7.31 (d, 2H, ArH: H2, H4 orto vs. CH_3_), 7.69 (d, 2H, ArH: H1, H5 orto vs. SO_2_). ^13^C NMR: δ 21.4 (1C), 24.8 (2C), 25.7 (1C), 32.9 (2C), 48.6 (1C), 127.1 (2C), 129.6 (2C), 136.8 (1C), 144.9 (1C), 153.1 (1C), 159.3 (1C).

*N–[(Z)–amino{[(4–chlorophenyl)carbamoyl]amino}methylidene]–4–methylbenzene–1–sulfonamide* (**p-TSA-c-e**) (Figure S5): ^1^H NMR (500 MHz): δ 2.32 (s, 3H, CH_3_), 6.5 (s, 2H, 2NH), 7.25 (s, 2H, NH_2_), 7.31–7.48 (m, 4H, ArH: H2, H4 orto vs. CH_3_; H20, H22 orto vs. Cl), 7.62–7.84 (m, 4H, ArH: H1, H5 orto vs. SO_2_; H19, H23 orto vs. NH). ^13^C NMR: δ 21.4 (1C), 121.6 (2C), 127.0–127.1 (3C), 129.0 (2C), 129.6 (2C), 130.8 (1C), 136.8 (1C), 144.9 (1C), 151.9 (1C), 159.3 (1C).

For the sulfonylurea derivatives **p-TSA-a** and **p-TSA-b**, the formation of the urea bridge is clearly evidenced in the ^1^H-NMR spectra by the presence of two distinct singlet signals for the secondary amine protons (-NH and -NHSO_2_) appearing between 6.50 and 7.52 ppm. In the ^13^C-NMR spectra of these compounds, the characteristic carbonyl resonance (C=O) is observed at 153.1 ppm (for **p-TSA-a**), providing unequivocal evidence of the urea bond formation.

A significant structural transition is observed in the hybrid sulfonylguanidine derivatives (**p-TSA-c-d** and **p-TSA-c-e**). In these cases, the ^13^C-NMR spectra exhibit two distinct downfield signals: one around 151.9–153.1 ppm, corresponding to the carbonyl carbon (C=O) of the urea moiety, and a second diagnostic signal at 159.3 ppm, assigned to the imino quaternary carbon (C=N) of the guanidine residue. This dual-signal pattern confirms the successful integration of the **p-TSA-c** intermediate into the final hybrid structure.

In the ^1^H-NMR spectra of the hybrid compounds, the symmetry of the p-substituted aromatic ring is maintained, evidenced by the characteristic doublets (J 8.0 Hz) for the ortho-protons relative to the –CH_3_ group (7.31 ppm) and the -*SO*_2_ group (7.69 ppm). The aliphatic regions for derivatives **p-TSA-a** and **p-TSA-c-d** clearly show the cyclohexyl protons as multiplets between 1.28 and 1.75 ppm, with the –CH proton attached to the nitrogen appearing further downfield at 3.87 ppm. The presence of the methyl group attached to the aromatic ring is consistently identified as a sharp singlet at 2.31–2.33 ppm in all spectra, with its corresponding carbon signal at 21.4 ppm.

### 2.3. Biological Activity

#### 2.3.1. Evaluation of the In Vitro Cytotoxicity of the New Compounds **p-TSA-a**, **p-TSA-b**, **p-TSA-c**, **p-TSA-c-d**, **p-TSA-c-e**

**Figure 2:**
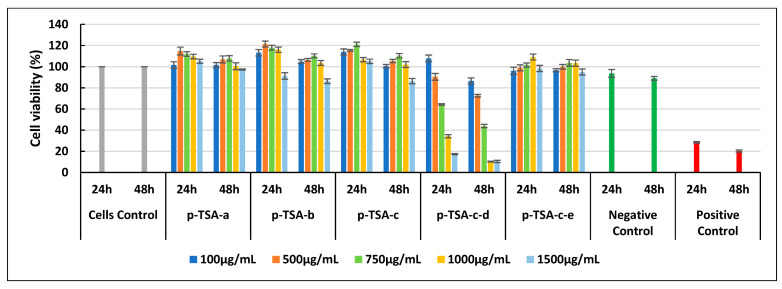
The cytotoxic profile of the studied compounds—determined in vitro in normal NCTC fibroblast cells using the MTT assay performed at 24 and 48 h intervals. The grey bars represent the untreated Cells Control (100% viability). The green bars represent the Negative Control, and the red bars represent the Positive Control. Different shades of blue, orange, green, and yellow correspond to the tested concentrations (100–1500 µg/mL) as indicated in the legend.

The graphic represents the cytotoxicity induced by samples on NCTC normal fibroblast cells by using the MTT assay, as shown in Figure 2.

In the in vitro evaluation on normal NCTC mouse fibroblast cells, the **p-TSA-a**, **p-TSA-b**, **p-TSA-c**, and **p-TSA-c-e** compounds demonstrated excellent biocompatibility, showing no cytotoxic effects across the entire concentration range of 100–1500 µg/mL; at both 24 and 48 h, they stimulated fibroblast proliferation, with cell viability values predominantly exceeding 105%. At the 24 h testing, **p-TSA-b** at 500 µg/mL and **p-TSA-c** at 750 µg/mL induced the highest proliferation rates, reaching maximum viability levels with values above 121%. The exception among the evaluated compounds was **p-TSA-c-d**, which, although biocompatible at 100 µg/mL, exhibited progressively increasing cytotoxicity beginning at 500 µg/mL. Consequently, within the 750–1500 µg/mL concentration range, **p-TSA-c-d** displayed pronounced cytotoxic effects, with cell viability declining from 43.84% to as low as 10.40%.

To provide a quantitative measure of the safety profile, the IC_50_ values were calculated for each derivative and observed in Table 3. Consistent with the high cell viability observed at 100 μg/mL, the calculated IC_50_ values significantly exceed the concentrations used for therapeutic evaluation.

The results show that all compounds possess very low toxicity, with IC_50_ values ranging from 772 μg/mL to >1500 μg/mL. These findings strongly support the high biocompatibility of the synthesized **p-TSA** derivatives at their therapeutically relevant concentrations and demonstrate an outstanding safety profile for the entire series.

**Figure 3:**
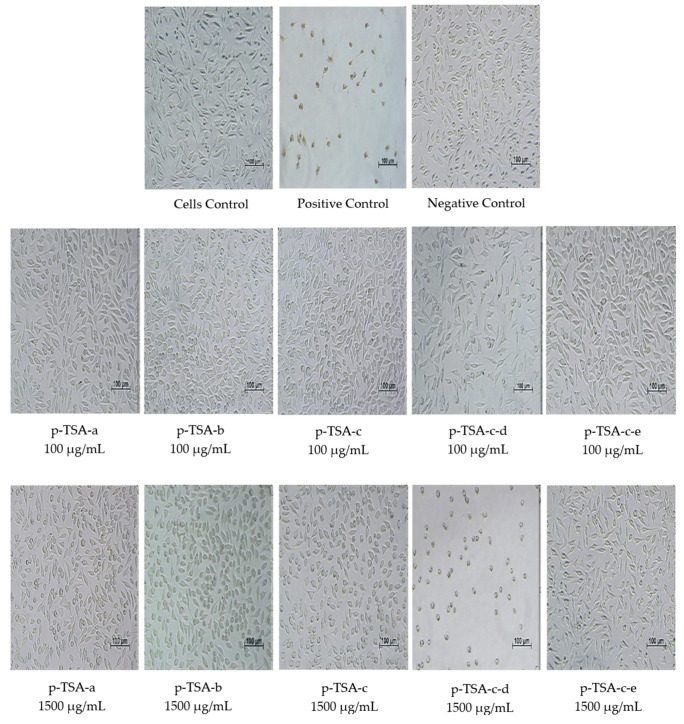
Images of morphology of NCTC cells after treatment, at 48 h of testing: Cells Control; Negative Control—0.2 g/mL HDPE extract; Positive Control—0.3% phenol solution; cells treated with **p-TSA** compounds at concentration of 100 and 1500 μg/mL, respectively.

In accordance with Figure 3, the Cells Control of normal NCTC mouse fibroblasts exhibits a characteristic morphology defined by elongated or spherical cells, each containing a centrally positioned nucleus with two to three nucleoli. The cytoplasm displays a finely granular texture, and the culture remains morphologically homogeneous at confluence, as observed at 48 h of incubation.

The aspect of the morphology of NCTC fibroblasts exposed to the Positive Control with 0.3% phenol solution demonstrates the pronounced cytotoxicity exerted by phenol. This treatment results in extensive disruption of the cellular substrate, including compromised cell membrane integrity, vacuolization of the intracytoplasmic content, and advanced nuclear degradation. Consequently, the overall fibroblast morphology becomes markedly altered, accompanied by a noticeable reduction in cell size.

At the 48 h evaluation, the morphological assessment of NCTC fibroblasts treated with the tested compounds at 100 µg/mL revealed cells with preserved characteristics and elongated or spherical forms; it also revealed the homogeneous aspect of the cell culture and that cell density was even higher than in the Cells Control, with the exception of **p-TSA-c-d**, for which a slight reduction in cell density was observed. These findings indicate that the compounds exhibited no cytotoxic effects at this concentration. In contrast, NCTC fibroblasts exposed to **p-TSA-c-d** compound at 1500 µg/mL displayed marked morphological alterations consistent with a strong cytotoxic response. The remaining adherent cells showed pronounced membrane disruption and loss of intracellular content, while numerous apoptotic cells had detached and migrated away from the substrate.

#### 2.3.2. Antidiabetic Activity

**Figure 4:**
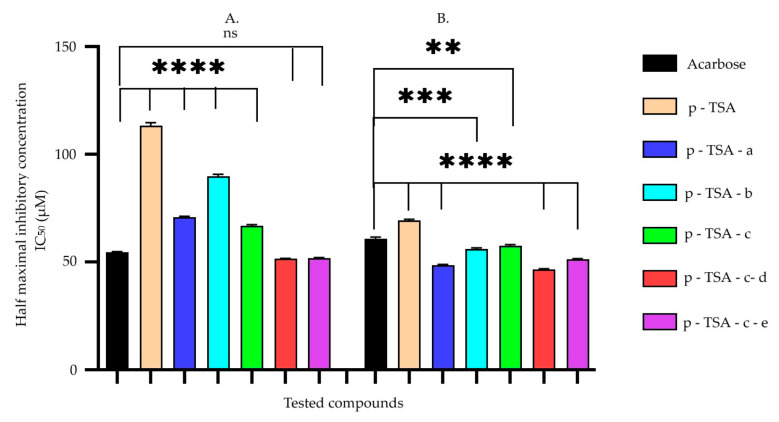
Comparative IC_50_ values against (**A**) α-amylase and (**B**) α-glucosidase inhibition activity of the synthetic compounds, using acarbose as a reference drug: ** *p* < 0.01, *** *p* < 0.001, **** *p* < 0.0001, ns—not significant, compared to acarbose, one-way repeated measures ANOVA (one-way ANOVA).

Based on in vitro studies, the results obtained by the synthetic compounds were compared with those of acarbose, which is a standard antidiabetic drug, but also with glycyclamide (**p-TSA-a**), an established reference drug. The biological evaluation was performed by inhibiting α-amylase and α-glucosidase enzymes and was completed by calculating the IC_50_. All sulfonamide derivatives were evaluated for their in vitro α-amylase and α-glucosidase inhibitory activities by determining their half-maximal inhibitory concentration (IC_50_) values compared to acarbose as a reference antidiabetic drug. The results are illustrated in Figure 4.

Regarding the inhibitory activity against α-amylase, a clear correlation between the structural complexity of the compounds and their biological potency was observed, in agreement with Figure S6. The precursor **p-TSA** exhibited the lowest activity, characterized by the highest IC_50_ value (113.29 ±4.12 μM), whereas its derivatization led to a progressive improvement in performance. The biuret-type compounds, **p-TSA-c-d** (red) and **p-TSA-c-e** (purple), demonstrated the lowest IC_50_ values in the series (51.47 ±0.68 μM and 52.01 ±1.36 μM, respectively), closely approaching the efficiency threshold of the reference drug acarbose (54.55 ±1.26 μM). This trend suggests that chain extension and the introduction of hydrophobic moieties (cyclohexyl and p-chlorophenyl) facilitate a more stable interaction with the enzyme’s active site, significantly reducing the concentration required for inhibition.

The results of the α-glucosidase assays highlight an even more promising activity profile, particularly for the cyclohexyl derivative, as also highlighted in Figure S7. Compound **p-TSA-c-d** (red) stands out for its remarkable potency, achieving an IC_50_ value lower than that of the reference drug, acarbose (61.30 ±0.70 μM vs. 46.54 ±0.72 μM, respectively). This observation indicates that the presence of the cyclohexyl moiety within the biuret structure provides superior binding affinity toward this specific enzymatic target. Furthermore, compounds **p-TSA-a** and **p-TSA-c-e** showed competitive IC_50_ values below the 60 μM threshold (49.14 ±1.48 μM and 51.30 ±1.45 μM), demonstrating that all synthetic modifications performed generated significantly more effective inhibitors than the parent molecule **p-TSA** (69.39 ±1.09 μM). The high statistical significance (*p* < 0.0001) compared to the control confirms the relevance of these novel structures as potential antidiabetic agents.

### 2.4. In Silico Analysis on the Interaction Between Enzymes and the Synthesized **p-TSA** Derivatives

**Figure 5:**
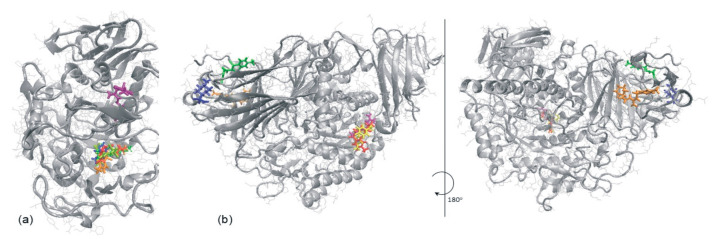
Superposition of the top scoring complexes resulting from the docking procedure when using the α-amylase (**a**) and α-glucosidase (**b**) as receptor for **p-TSA** (blue), **p-TSA-a** (red), **p-TSA-b** (green), **p-TSA-c** (purple), **p-TSA-c-d** (orange), and **p-TSA-c-e** (yellow). The enzymes and ligands are represented in New Cartoon and Licorice style, respectively, by means of the Visual Molecular Dynamics 1.9.3. software.

**Figure 6:**
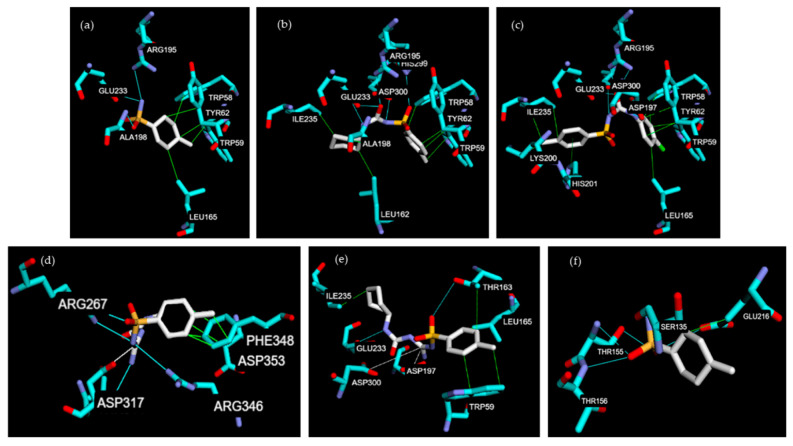
Atomic-level details on the interactions between α-amylase and **p-TSA** (**a**), **p-TSA-a** (**b**), **p-TSA-b** (**c**), **p-TSA-c** (**d**), **p-TSA-c-d** (**e**), and **p-TSA-c-e** (**f**). Hydrogen bonds are hydrophobic interactions and are represented in cyan and lime, respectively.

**Figure 7:**
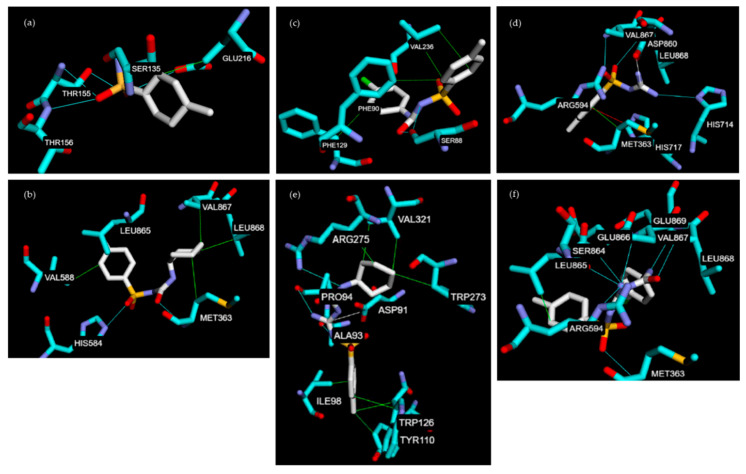
Atomic-level details on the interactions between α-glucosidase and **p-TSA** (**a**), **p-TSA-a** (**b**), **p-TSA-b** (**c**), **p-TSA-c** (**d**), **p-TSA-c-d** (**e**), and **p-TSA-c-e** (**f**). Hydrogen bonds are hydrophobic interactions represented in cyan and lime, respectively.

In order to gather atomic-level details on the interaction between the two enzymes responsible for carbohydrate hydrolysis in the digestive system and the **p-TSA** derivatives developed in our work, molecular docking tests were performed. For each of the twelve investigated enzyme–ligand complexes, the top scoring three-dimensional model was checked in-depth, to identify potential ligand binding interference with the hydrolytic activity of the enzyme. The 3D models of the enzyme–ligand complexes as well as the legal site of the models with maximum scores are presented in Figure 5, Figure 6 and Figure 7, while Table 4 reports the results of the binding energy, the interaction area and the amino acids involved in the different types of interactions with the ligand.

Based on the binding energy values presented in Table 4, we can observe that α-amylase exhibited better affinity towards all tested ligands, compared to α-glucosidase. Thus, it appears that α-amylase has the highest affinity towards **p-TSA-c-d** and **p-TSA-c-e** (binding energies of −8.8 and −8.4 kcal/mol, respectively), whereas the lowest affinity was observed in case of the complex with **p-TSA** (binding energy of −5.8 kcal/mol). The interface area of the α-amylase-based complexes varied between 149.6 and 234.5 Å2, observed in case of **p-TSA-c** and **p-TSA-b**, respectively.

Among all tested ligands, α-glucosidase appears to exhibit the best affinity towards **p-TSA-c-d** (binding energy of −8.2 kcal/mol), with the interaction between the two molecules consisting of eight hydrophobic contacts, three hydrogen bonds and one salt bridge. As in case of α-amylase, α-glucosidase appeared to have the lowest binding affinity towards **p-TSA** (binding energy of −5.4 kcal/mol). In case of the complexes formed with α-glucosidase, the lowest and highest interface areas of 151.1 Å2 and 225.4 Å2 were measured in the case of **p-TSA-c-d** and **p-TSA-b**, respectively.

The interaction between α-amylase and the tested ligands mainly relies on hydrophobic contacts and hydrogen bonds (Table 4). The highest number of contacts involving the amino acids located on the α-amylase surface were observed when the docking was performed with **p-TSA-b** (nine hydrophobic interactions, five hydrogen bonds and one Π-stacking), followed by **p-TSA-a** (seven hydrophobic interactions and five hydrogen bonds) and **p-TSA-c** (five hydrophobic interactions, four hydrogen bonds and one salt bridge). It should be noted that, except for **p-TSA-c**, all tested ligands established hydrophobic contacts with the Trp^58^, Trp^59^ and Tyr^62^ residues of α-amylase. Moreover, except for **p-TSA-c-e**, ligand binding occurred in the close vicinity of the enzyme active site.

Also, in the case of α-glucosidase, the interaction between the enzyme and the tested ligands mainly relies on hydrophobic contacts and hydrogen bonds (as shown in Table 4). In this case, the highest number of contacts involving the amino acids located on the α-glucosidase surface was observed when the docking was performed with **p-TSA-c-d** (eight hydrophobic interactions, three hydrogen bonds and one salt bridge), followed by **p-TSA-c-e** (one hydrophobic interaction and eight hydrogen bonds) and **p-TSA-a** (six hydrophobic interaction and two hydrogen bonds).

## 3. Discussion

Used for a long time for chronic conditions such as type 2 diabetes mellitus, sulfonamides have been of interest for this study due to their advantages, with p-toluenesulfonamide being the protagonist. To obtain new synthetic agents, condensation with a series of thioisocyanates was chosen, which are of particular interest to researchers due to their role as fundamental compounds in various multicomponent reactions, which have led to the development of important new molecules [25]. For example, researchers Lotfi M. Aroua et al. used a series of different isocyanates (phenylisocyanate, 4-methoxyphenylisocyanate, cyclohexylisocyanate, isopropyl isocyanate, butyl isocyanate, 1,4-phenylene diisocyanate, and 1,4-bis(isocyanatomethyl)cyclohexane) for the synthesis of benzimidazole-urea [27].

In the present study, the spectral characterization of the new derivatives was conducted using two spectrometric methods: NMR and ATR-FTIR. Thus, the presence confirmation of sulfonamide, amide, azomethine, and aromatic halogenated compounds, provides the validation of the newly synthesized compounds. For example, signals at 2.32 ppm within the ^1^H-NMR spectrum confirm the presence of methyl protons (CH_3_). Similar observations have been reported in the literature for structurally related compounds [28]. The multiplets at 1.36, 1.75, and 3.87 ppm were assigned to the CH_2_ (methylene) protons in the cyclohexyl ring structure and CH (methine) protons, respectively [29]. Similarly, the singlets at 6.5 and 7.52 ppm were assigned to NH (amide) protons, as reported in the literature [30]. The doublets at 7.29 confirm the presence of NH_2_ (amine) protons, while aromatic protons appeared in the range 7.31–7.32, 7.69–7.71 ppm. Additionally, physicochemical analyses were performed to evaluate the quality of the compounds and key parameters such as the chemical structure, the yield, the melting point, and the solubility were determined. The relationship between the structure and antidiabetic activity of the derivatives is highlighted by the various functional groups present in each compound, including the azomethine group, amide group, heterocyclic group, and aromatic groups.

In a cytotoxicity test, biocompatibility assessment on the NCTC cell line demonstrated that most **p-TSA** derivatives (**a**, **b**, **c**, and **c–e**) possess an excellent toxicological profile, a critical result for compounds with potential chronic administration in diabetes management. Biocompatibility maintained up to concentrations of 1500 µg/mL, which places these derivatives in a safety zone superior to many experimental antidiabetic agents reported in the literature, which often exhibit cytotoxicity at much lower doses [31,32,33]. Furthermore, the ability of **p-TSA-b** and **p-TSA-c** compounds to stimulate fibroblast proliferation (viability > 121%) represents a significant collateral therapeutic advantage. The literature frequently emphasizes that chronic hyperglycemia impairs fibroblast function, leading to delayed wound healing in diabetic patients [34]. Thus, a sulfonamide compound that not only lowers blood glucose but also stimulates the metabolic activity of fibroblasts could offer dual benefits, unlike classic sulfonylureas (e.g., glibenclamide), which in some in vitro studies have shown a neutral or slightly inhibitory effect on cell proliferation at high concentrations [35]. The pronounced toxicity of the **p-TSA-c-d** derivative (decrease to 10.40%) is consistent with SAR (structure–activity relationship) observations, indicating that certain modifications of the sulfonamide nucleus can induce apoptosis through oxidative stress or mitochondrial potential disruption [36]. This drastic decrease in viability suggests that, although the **p-TSA** skeleton is generally well-tolerated, the specific substitution in the **p-TSA-c-d** compound generates a pharmacotoxic profile that excludes it from use as a systemic antidiabetic agent, unlike the other derivatives, which confirm their safety for use in wide therapeutic windows [37].

Numerous studies that have been reported in international databases have addressed the topics of development and description of chemical syntheses, starting from sulfonamides and aiming to obtain new derivatives that act as antidiabetics, inhibiting α-amylase and α-glucosidase enzymes [38,39,40]. The p-substituted sulfonamide ring, having a nitrogen atom attached, which in turn is attached to a phenyl, cyclic or heterocyclic ring, is a common structural feature of sulfonamide compounds, such as the **p-TSA-a**, **p-TSA-b**, **p-TSA-c-d**, **p-TSA-c-e** compounds, which have been in vitro evaluated for their α-glucosidase and α-amylase enzyme inhibitory activities.

Thus, in the experiment of the present study, new compounds with a sulfonamide structure were synthesized and evaluated in vitro for their antihyperglycemic effect compared to the standard drug, acarbose. The synthesized compounds showed IC_50_ values ranging from 51.47 to 113.29 μM, with **p-TSA-c-d** and **p-TSA-c-e** being the compounds with the strongest α-amylase inhibition potential. This aspect is closely related to their chemical structure and the presence of the two residues of cyclohexylisocyanate and chlorophenylisocyanate, respectively, inserted into the sulfonamide structure, by means of a urea residue. This intermediate step with urea favors the obtaining of more potent compounds, compared to the direct binding in the case of the compounds **p-TSA-a** and **p-TSA-b**. Compared to acarbose, **p-TSA-c-d** and **p-TSA-c-e** compounds are 1.05 and 1.04 times, respectively, more active.

Regarding α-glucosidase inhibitory activity, the most prominent sulfonamide derivatives, namely **p-TSA-c-d**, followed by **p-TSA-a** and **p-TSA-c-e**, showed excellent inhibitory potential with IC_50_ values of 46.54 μM, 49.14 μM, and 51.3 μM, respectively. Additionally, this time, the two residues of cyclohexylisocyanate and chlorophenylisocyanate, by means of a urea residue, have left their mark on the synthesized compounds, demonstrating the high potency of these compounds. Thus, compared to acarbose, they were 1.24 to 1.31 times more potent. The compounds **p-TSA-b** and **p-TSA-c** showed moderate α-glucosidase inhibition activity with IC_50_ in the range from 56.08 μM to 57.69 μM.

Similar studies carried out on sulfonamide compounds have been reported in the literature to date. For example, in the research for a novel sulfonamide compound, Mohammed Salah Ayoup M.S. et al. evaluated the enzymatic activity of 15 new derivatives, where only four different hydrazinyl–benzenesulfonamide obtained satisfactory results for α-glucosidase inhibition relative to the standard acarbose (19.39, 25.12, 25.57 and 22.02 μM). Regarding α-amylase inhibition activity, all compounds obtained lower percentages due to weak interactions with this enzyme (in the range of 57.36–416.46 μM), which demonstrates that the new synthetic compounds developed in our study exhibit superior α-amylase inhibitory activity [41].

In another study reported by Apaydin et al., the antidiabetic activity has been evaluated in vitro by α-glucosidase and α-amylase inhibition tests of new hydrazone sulfonamide derivatives. In this study, significant results were obtained in the case of α-glucosidase inhibition activity, where the IC_50_ value of 65.27 µg/mL (corresponding to 165.02 µM) was obtained for the synthetic hydrazone, with this compound being much active than the standard acarbose with an IC_50_ value of 122.25 µg/mL (corresponding to 189.35 µM) [38]. Woo Duck Seo et al. reported new 20 sulfonamide–chalcone hybrid compounds, where 12 synthetic agents did not have α-amylase inhibitory activity, four were strong inhibitors against α-amylase (with IC_50_ values at 37.3 μM and 193.7 µM), and four had mild inhibitory activity against *β*-amylase (126.8–206.5 µM) and no inhibition for α-amylase. Against α-glucosidase, only sulfonamide chalcone 20 showed stronger inhibitory activity than acarbose (IC_50_ = 60.8l μM) [42].

In addition, molecular docking tests were employed in the present study to find out the potential mechanism involved in the inhibitory ability of **p-TSA** derivatives on α-amylase and α-glucosidase. The in-depth analysis of the top scoring complexes revealed that, except for **p-TSA-c**-**e**, all investigated ligands directly bind in the vicinity of the active site of α-amylase, therefore potentially interfering with the recognition of the starch (specific substrate of α-amylase) and its transformation. In particular, the compound **p-TSA-c-e** has the most voluminous chemical structure and the highest molecular mass, which provides a different spatial orientation than the other compounds and, as a result, a weaker interaction with the enzyme. In the meantime, the **p-TSA**, **p-TSA-a**, **p-TSA-b** and **p-TSA-c-d** molecules were able to establish hydrogen bonds with the Glu^233^ residue located in a sub-pocket of the α-amylase-active site^30^. An additional hydrogen bond with Asp^300^, a second residue of the same patch of the active site^30^, was observed in the complexes with **p-TSA-a** and **p-TSA-b** (Table 3). Finally, in case of the α-amylase **p-TSA-c-d** complex, a salt bridge involving the Asp^300^ reside was observed as well, contributing to the low binding energy value which suggests a good affinity towards the active site. The best fits predicted when using the α-glucosidase as receptor in the docking procedure indicated that all **p-TSA** derivatives were attached to the enzyme surface, establishing various types of noncovalent interactions with the amino acids of their biding pockets. Since no ligand was found in direct contact with the active site of the α-glucosidase, one might consider that the inhibitory activity observed experimentally might be due to allosteric transitions triggered by ligands binding to various allosteric sites on the protein surface [43].

While targeted docking is the standard for known active sites, we employed the Blind Docking Server to perform an unbiased search across the entire protein surface. This approach was intended to explore whether our **p-TSA** derivatives might exert their inhibitory effect through allosteric modulation or secondary binding sites, which are sometimes overlooked in conventionally targeted docking. Despite the inherent limitations of blind docking algorithms, the binding poses identified for the **p-TSA-c-d** derivative showed a strong correlation with our in vitro enzymatic assays. The high binding affinity scores predicted by the Blind Docking Server reflect the low IC50 values obtained experimentally, suggesting that the identified interactions are biologically relevant. We acknowledge that newer AI-based docking frameworks and targeted tools like AutoDock Vina may offer higher sampling precision. However, within the scope of this preliminary structural evaluation, our current findings provide a consistent molecular rationale for the observed inhibitory activity of the synthesized sulfonamides. The architectural design of our **p-TSA** derivatives aligns with the pharmacophoric model recently proposed by Ranade et al., who synthesized a series of sulfonamide-thiazolidin-4-one hybrids as potent α-amylase inhibitors. Their comprehensive study, which combined DFT calculations and molecular dynamics, demonstrated that the benzenesulfonamide core is indispensable for establishing stable interactions within the enzyme’s binding pocket [44]. Our findings reinforce this observation, as the **p-TSA**-based scaffolds similarly targeted the catalytic residues, yielding a consistent inhibitory profile and providing further evidence for the antidiabetic potential of this class of compounds.

Regarding the correlation between in vitro and in silico results, we can note that in silico α-amylase has the highest affinity towards **p-TSA-c-d** and **p-TSA-c-e**, which correlates with the lowest IC_50_ values identified in vitro for these compounds. Also, in the case of α-glucosidase, the in silico results revealed the highest affinity for **p-TSA-c-d**, which correlates with the lowest IC_50_ value identified in vitro for this compound.

The structure–activity relationship (SAR) analysis of the synthesized derivatives reveals that the biological potency is significantly modulated by the nature of the bridge between the **p-TSA** scaffold and the terminal hydrophobic moieties. In the case of **p-TSA-a** and **p-TSA-b**, the presence of a classic sulfonylurea bridge facilitates hydrogen bonding with the enzyme’s active site. However, the superior inhibitory potential observed for **p-TSA-c-d** suggests that the transition to a sulfonylguanidine–urea hybrid system (introduced via intermediate **p-TSA-c**) enhances binding affinity. This may be attributed to the increased basicity and nitrogen density of the guanidine-like moiety, which provides additional electrostatic interactions and resonance stabilization. Furthermore, the substitution with a cyclohexyl ring (derivative **p-TSA-c-d**) vs. a p-chlorophenyl ring (derivative **p-TSA-c-e**) highlights the importance of lipophilicity and steric fit; the saturated cyclohexyl system likely optimizes hydrophobic contacts within the enzymatic pocket more effectively than the electron-withdrawing p-chlorophenyl group, leading to the enhanced biological response observed in this series.

Therefore, the strategic modification of the **p-TSA** scaffold through the introduction of sulfonylguanidine–urea moieties has yielded a series of potent enzymatic inhibitors, among which **p-TSA-c-d** emerges as a noteworthy scaffold. The synergistic correlation between its superior α-amylase and α-glucosidase inhibitory activity, favorable binding energy within the catalytic pockets, and its low cytotoxicity profile highlights the potential of these derivatives as safe and effective scaffolds for the development of next-generation oral antidiabetic agents.

## 4. Materials and Methods

### 4.1. Synthesis of **p-TSA** Derivatives

The chemical agents involved in the chemical synthesis were used in pure state and purchased from Sigma-Aldrich, represented by 99% **p-TSA**, 98+% cyclohexyl isocyanate, 98% 4-chlorophenyl isocyanate, 99.98% urea, potassium carbonate catalyst, acetone and 10% hydrochloric acid (HCl). For this step, a reflux system (Biobase Biodustry, Shandong, China) and a POL-EKO SLN 53 oven STD (Wodzislaw Slaski, Poland), have been used.

#### 4.1.1. Synthesis of N-(cyclohexylcarbamoyl)-4-methylbenzenesulfonamide (C_14_H_20_N_2_O_3_S, **p-TSA-a**, Glycyclamide) and N-[(4-chlorophenyl)carbamoyl]-4-methyl- benzene-1-sulfonamide (**p-TSA-b**)

The preliminary synthesis of one standard was intended to refine the method of obtaining further compounds and for subsequent use in in vitro enzyme evaluation. Thus, according to the protocol developed by Sadineni et al. for the synthesis of glycyclamide, **p-TSA** (1 g) was dissolved in acetone (10 mL) to which cyclohexyl isocyanate (**a**) and the catalyst K_2_CO_3_ was added in a molar ratio of 1:1.2:1.1 [45]. The mixture was refluxed for 2 h at 85 °C, resulting in a white precipitate. At the end of refluxing, the product obtained was collected through filtration, purified through recrystallization in ethanol and then dried in an oven at 60 °C. The resulting compound was named **p-TSA-a**, respectively, N-(cyclohexylcarbamoyl)-4-methylbenzenesulfonamide or glycyclamide. Using the same molar ratio and conditions, **p-TSA** (1 g) was also condensed with 4-chlorophenyl isocyanate (**b**) for a total reflux time of 2 h, resulting in a white precipitate. The synthesis product obtained was named **p-TSA-b**, respectively, N-[(4-chlorophenyl)carbamoyl] -4-methylbenzene-1-sulfonamide.

#### 4.1.2. Synthesis of N-(diaminomethylidene)-4-methylbenzene-1-sulfonamide (**p-TSA-c**), N-{amino[(cyclohexylcarbamoyl)amino]methylidene}-4-methylbenzene-1-sulfonamide (**p-TSA-c-d**) and N-[(Z)-amino{[(4-chlorophenyl)carbamoyl]amino}methylidene]-4-methylbenzene-1-sulfonamide (**p-TSA-c-e**)

Condensation of **p-TSA** with (**c**) urea, in the presence of anhydrous K_2_CO_3_, was carried out on a 1:1.2:1.1 molar ratio. Refluxing was carried out in acetone solvent (30 mL); after 4 h of refluxing the product **p-TSA-c**, N-(diaminomethylidene)-4-methylbenzene-1-sulfonamide was obtained as a white precipitate, which was filtered and dried afterwards. This synthetic compound, studied in a previous published paper by our group [46], served as a starting point for obtaining the following sulfonamide derivatives. Therefore, **p-TSA-c** reacted with (**a**) cyclohexyl isocyanate in acetone medium (10 mL) after a reflux time of 4 h at 85 °C, in the presence of the same catalyst. The resulting product, a white precipitate, was renamed **p-TSA-c-d**, i.e., N-{amino[(cyclohexyl carbamoyl)amino]methylidene}-4-methylbenzene-1-sulfonamide. Another amount of the **p-TSA-c** product was used for the reaction with (**b**) 4-chlorophenyl isocyanate under the same conditions mentioned above, maintaining the same molar ratio, thus obtaining the **p-TSA-c-e** derivative, respectively, N-[(Z)-amino{[(4-chlorophenyl)carbamoyl] amino}methylidene]-4-methylbenzene-1-sulfonamide. In each case, the remaining residue (containing unreacted K_2_CO_3_) was treated with ultrapure distilled water and acidified with 10% HCl to remove the catalyst as an impurity from the final reaction products.

### 4.2. Characterization and Structural Confirmation of **p-TSA** Derivatives

#### 4.2.1. Physicochemical Properties

The physicochemical stability and solubility of the 5 mg/mL synthetic agents in various solvents were tested: deionized water, 99.98% acetone, 99.5% DMSO, min 99.5% dimethylformamide (DMFA), ethyl alcohol, ethyl alcohol 50°, dioxane, toluene, 100 g/L sodium hydroxide, ethyl acetate, dichloroethane, isopropyl alcohol, methyl alcohol. The melting points of the compounds were measured using the STUART SMP 10 digital apparatus (UK). The Advanced Chemistry Development (ACD/ChemSketch, version 11.02) software was used to analyze and produce the structural formula, and to identify the molecular formula and relative masses. Based on the practical quantities obtained, the reaction yield was calculated for each compound.

#### 4.2.2. ATR-FTIR Spectral Analysis

ATR-FTIR (Attenuated Total Reflexion–Fourier Transform Infrared Spectroscopy) analysis was performed using the Cary 630 KBr Engine spectrometer (Agilent Technologies, Petaling Jaya, Malaysia) controlled by the MicroLab B. 05.6 software, in the range 4000–500 cm^−1^, in transmittance mode. Crystal cleaning was performed with isopropyl alcohol, and the spectra were recorded after 32 scans, at a resolution of 8 cm^−1^. The spectra processing was carried out with the Horizon MBTM FTIR Software.

#### 4.2.3. ^1^H-NMR and ^13^C-NMR Spectroscopy

^1^H-NMR and ^13^C-NMR (Nuclear Magnetic Resonance) spectra were recorded using a Bruker Avance III instrument at 500 MHz in deuterated dimethylsulfoxide (DMSO-*d_6_*). The shift in the chemical signals present in the recorded spectra was expressed in δ values expressed in parts per million (ppm), and the coupling constants *J* were expressed in Hz, in the 3–300 GHz radio frequency region. The chemical identity and purity of the precursors of **p-TSA** were confirmed by the Vanquish Flex UHPLC system coupled with a high-resolution Orbitrap Exploris 120 mass spectrometer (Thermo Fisher Scientific, Waltham, MA, USA) data with those previously reported in our earlier study [46].

### 4.3. Cytotoxicity of Samples

#### 4.3.1. Cell Lines and Culture Conditions

Mouse fibroblasts NCTC clone 929 were acquired from the European Collection of Authenticated Cell Cultures (ECACC, UK). The cells were cultured in Minimum Essential Medium (MEM) supplemented with 10% Ser Fetal Bovin (FBS) and 1% penicillin–streptomycin–neomicin. The cell culture was grown as an adherent monolayer at 37 °C in a humidified incubator with 5% CO_2_. Cells were subcultured upon reaching 85–90% confluence.

#### 4.3.2. MTT Assays

For cytotoxicity determination, the NCTC cells were seeded in 96-well plates at a density of 4000 cells in 100 µL medium per well and incubated in for 24 h under specific conditions. Sample stock solutions of concentration 1500 μg/mL were prepared in culture medium. After 24 h of cell incubation, in the subconfluent stage of cell culture, the medium in the wells assigned to Cells Control was replaced with fresh medium, and the sample solutions at the specified concentrations of 100, 500, 750, 1000 and 1500 µg/mL were added to the corresponding wells, while two test controls of experiment were distributed to their designated wells, namely a Negative Control consisting of HDPE (High-Density Poly Ethylene) granules extract 0.2 g/mL and a Positive Control of 0.3% phenol solution. The in vitro cell culture experiment was conducted in three independent biological replicates. Each sample concentration was evaluated in triplicate.

At the two intervals of 24 and 48 h, the viability of treated cells was assessed using the (3-[4,5-dimethylthiazol-2-yl]-2,5 diphenyl tetrazolium bromide (MTT) assay by replacing the solutions in the wells with a 50 µg/mL MTT solution, followed by a 3 h incubation under the previously described conditions. The MTT solution was then removed and replaced with isopropanol to solubilize the resulting formazan crystals—a step performed with orbital shaking for 15 min. Absorbance was measured at 570 nm using a Spectro Star BMG spectrophotometer (Labtech, Ortenberg, Germany). The viability percentage of treated cells with samples is calculated as being their optical density reported to optical density of untreated control cells, where the value of Cells Control is considered as 100% [47].

### 4.4. In Vitro Evaluation of Antidiabetic Activity

The antidiabetic activity of the synthesized compounds was evaluated by inhibitory tests of the main enzymes responsible for carbohydrates hydrolysis, such as α-amylase from *porcine pancreas* (E.C. 3.2.1.1, type VI) and α-glucosidase from *Saccharomyces cerevisiae* (type I, >10 units/mg protein) enzymes purchased from Sigma-Aldrich. The percentage of inhibition was determined using Equation (1), while the IC_50_ (half-maximal inhibitory concentration) values were calculated from the dose–response curves generated by testing a range of standard concentrations for each compound: 13, 20, 27, 41, 55, and 110 μM. All enzymatic assays were performed in three independent biological replicates (*n* = 3) and the results are expressed as mean ± standard deviation (SD).

%I = (A_control_ − A_sample_)/A_control_ × 100, (1)

where I is percent of inhibition and A is absorbance

The methods employed were performed by using a UV-1900 spectrophotometer (Schimadzu, Columbia, MD, USA).

#### 4.4.1. α-Amylase Inhibition

The biological tests of α-amylase inhibition were performed according to the procedures reported by Jing Wang et al. [48]. The synthetic compounds were used to prepare 1% stock solutions dissolved in 0.1 M DMSO, which were subsequently diluted to obtain the final concentration range (13–110 μM) in the reaction mixture. To each 50 μL sample, 50 μL α-amylase (prepared in 0.02 M Na_2_CO_3_ buffer) was added. The samples were kept on ice for 5 min, then 50 μL of 1% starch solution was added and incubated for 20 min at 37 °C. To denature the enzyme and stop the reaction, 500 μL of 0.4 M hydrochloric acid (0.4 M HCl) solution, 50 μL iodine iodide solution (50 mM), and 2300 μL distilled water were added. Samples without enzyme were prepared according to the same working protocol, and a blank sample and a control sample were also prepared, with the absorbance at a wavelength of 600 nm being read, for all solutions. Acarbose and DMSO were employed as positive control and negative control, respectively.

#### 4.4.2. α-Glucosidase Inhibition

To evaluate the inhibitory potential of α-glucosidase by synthetic compounds, as well as by the standard acarbose, a previously reported protocol was adapted [49]. The assay utilized α-glucosidase (1 mg/mL), 100 mM phosphate buffer (PBS, pH 6.9), 2% acarbose, 0.2 M Na_2_CO_3_ and 25 mM p-nitrophenyl-α-D-glucopyranoside (PNPG) as the substrate. Serial dilutions of the synthetic compounds were prepared to achieve the same final concentrations (13, 20, 27, 41, 55, and 110 μM) in the assay volume. From these solutions, 100 μL aliquots were mixed with 50 μL of enzyme, and the solutions were left at room temperature (21 °C) for 10 min. Subsequently, 2000 μL of PBS and 50 μL of PNPG were added to initiate the reaction, followed by an initial incubation at 37 °C for 20 min. To stop the reaction, 0.2 M Na_2_CO_3_ (800 μL) was added. Absorbance was then measured using a spectrophotometer at a wavelength of 405 nm. A sample containing all reagents except the enzyme was analyzed under identical conditions to serve as a blank. Acarbose and DMSO were employed as positive control and negative control, respectively.

### 4.5. In Silico Tests on the **p-TSA** Derivatives Binding to Enzymes

The HyperChem 8.0 software (Hypercube, Inc., Ontario, Canada) was used to build and optimize the molecular models of the **p-TSA, p-TSA-a**, **p-TSA-b**, **p-TSA-c**, **p-TSA-c-d**, and **p-TSA-c-e** compounds synthesized in the laboratory scale experiment. The energy of the molecular models was minimized using a sequence of Steepest descent and Conjugate gradient algorithms, such as obtaining a final root mean square gradient of 0.0001 kcal/(Å·mol). The optimized models were further used as ligands for α-amylase and α-glucosidase receptors in the molecular docking procedure carried out by means of the Blind Docking Server [50].

Docking validation was performed through pose reproduction performed for both receptors used in the study. The heavy atom root mean square deviation (RMSD) values, calculated upon superimposing the complexes including either α-amylase or α-glucosidase as receptor, were lower than 1.5 Å, thus supporting the reliability of the docking results.

The three-dimensional models of the α-amylase [51] and α-glucosidase [52] were gathered from the RCSB Protein Data Bank. The top enzyme–ligand solutions were decided based on the protein surface screening method which allows identifying all hotspots existing on the entire surface of the receptors, that might be involved in accommodating small molecules. The resulting complex solutions were ranked based on the electrostatic forces, Van der Waals interactions and the hydrogen bonds established between molecules [50]. For each type of enzyme–ligand complex, detailed analysis of the top scoring model was finally carried out by means of Visual Molecular Dynamics [43], whereas the noncovalent interactions between the two molecules of each complex were calculated using the dedicated tools of PLIP v1.3.2. [53].

### 4.6. Statistical Analysis

Data were expressed as mean ± S.E.M. Multiple groups’ mean of parametric data sets were compared using one-way analysis of variance (ANOVA) after it was determined that the data conformed to a normal distribution. If an overall significance was found, a multiple-comparisons test was to be used with GraphPad Prism 10 (GraphPadSoftware Inc. San Diego CA, USA). The value of *p* < 0.05 was considered as significant.

## 5. Conclusions

This research successfully explored the antidiabetic potential of several novel sulfonamide derivatives. The condensation reactions of **p-TSA** with urea, cyclohexyl isocyanate, and 4-chlorophenyl isocyanate proceeded with satisfactory yields, and the resulting structures were unequivocally confirmed via ATR-FTIR and NMR spectroscopy.

The cytotoxicity study on NCTC fibroblasts confirmed a high safety profile for most of the synthesized derivatives. While compounds **p-TSA-a**, **p-TSA-b**, **p-TSA-c**, and **p-TSA-c-e** proved to be fully biocompatible across the entire concentration range (100–1500 µg/mL)—even stimulating cellular metabolic activity up to values of 121%—the **p-TSA-c-d** derivative exhibited a distinct behavior. The latter maintained a non-toxic character only at the minimum dose of 100 µg/mL, with severe cytotoxicity occurring at concentrations exceeding 750 µg/mL, where cell viability declined drastically to 10.40%. These findings underscore the critical role of structural modulations in balancing pharmacological efficacy with cellular safety.

In vitro biological assays demonstrated that the synthesized derivatives possess significant inhibitory activity against both α-amylase and α-glucosidase. In the α-amylase inhibition assays, the compounds exhibited robust potency with IC_50_ values comparable to the acarbose standard. Most notably, the biuret and sulfonamide derivatives showed exceptional performance against α-glucosidase. Specifically**, p-TSA-c-d,** followed by **p-TSA-a** and **p-TSA-c-e**, significantly outperformed acarbose, exhibiting 1.24 to 1.31 times higher potency. These findings highlight **p-TSA-c-d** as a noteworthy scaffold, suggesting that the extension of the sulfonylurea system into a biuret framework represents a potential strategy for developing new carbohydrate-digestive enzyme inhibitors capable of controlling postprandial hyperglycemia. The molecular docking tests revealed different mechanisms for the inhibitory activity exerted by the **p-TSA** derivatives on the α-amylase and α-glucosidase enzymes. In conclusion, the results obtained for aryl–sulfonamide derivatives represent a new class of strong amylase and glycosidase inhibitors and provide promising directions for both in vitro and in vivo evaluation studies, thus leading to new research opportunities in the treatment of diabetes mellitus.

## Figures and Tables

**Table 1:** Physicochemical characteristics of the obtained compounds.

Compound	Molecular Formula	Molecular Weight (g/mol)	Aspect	Melting Point (°C)
p-TSA *	C_7_H_9_NO_2_S	171.2168	white amorphous powder	139
**p-TSA-a**	C_14_H_20_N_2_O_3_S	296.3852	silky white acicular crystals	182
**p-TSA-b**	C_14_H_13_ClN_2_O_3_S	324.7826	white crystalline powder	177
**p-TSA-c**	C_8_H_11_N_3_O_2_S	213.2568	white prismatic crystals	192
**p-TSA-c-d**	C_15_H_22_N_4_O_3_S	338.4251	white acicular crystals	179
**p-TSA-c-e**	C_15_H_15_ClN_4_O_3_S	366.8226	white prismatic crystals	185

* Bold identifiers refer to the synthesized compounds according to Scheme 1.

**Table 2:** Solubility at 5 mg/mL of the obtained compounds and **p-TSA** in various solvents.

Solvents	Soluble	Partial Soluble	Insoluble
Distilled water	4	1, 3, 6	2, 5
Ethyl alcohol	4	1, 2, 3, 5, 6	-
Methyl alcohol	2, 4, 5, 6	1, 3	-
Isopropyl alcohol	-	1, 3	2, 4, 5, 6
Acetone	1, 2, 3, 4, 5, 6	-	-
Ethyl acetate	5	1, 2, 3	4, 6
DMSO	1, 2, 3, 4, 5, 6	-	-
DMFA	1, 2, 3, 4, 5, 6	-	-
Dioxane	-	-	1, 2, 3, 5, 6
Toluene	3	1	1, 2, 4, 5
Dichlorethane	-	3	1, 2, 4, 5, 6

1—**p-TSA**; 2—**p-TSA-a**; 3—**p-TSA-b**; 4—**p-TSA-c**; 5—**p-TSA-c-d**; 6—**p-TSA-c-e**.

**Table 3:** Cytotoxic activity (IC_50_ values) of the synthesized compounds.

Compound	p–TSA-a	p–TSA-b	p–TSA-c	p–TSA-c-d	p–TSA-c-e
IC_50_ (μg/mL)	>1500	>1500	>1500	772	>1500

**Table 4:** Details on the single-molecule-level interactions within the top scoring enzyme–ligand complexes resulting in the docking procedure.

	Ligands					
	p-TSA	p-TSA-a	p-TSA-b	p-TSA-c	p-TSA-c-d	p-TSA-c-e
α-amylase–ligand complexes	Amino acids in contact with the ligand					
Hydrophobic interactions	Trp^58^, Trp^59^, (2) Tyr^62^, Leu^165^	Trp^58^, (2) Trp^59^, Tyr^62^, Leu^162^, Ala^198^, Ile^235^	(2) Trp^58^, Trp^59^, (2) Tyr^62^, Leu^165^, Lys^200^, (2) Ile^235^	(4) Phe^348^, Asp^353^	(2) Trp^59^, Thr^163^, Leu^165^, Ile^235^	Trp^58^, Trp^59^, (4) Tyr^62^, Leu^162^
Hydrogen bonds	Arg^195^, Ala^198^, Glu^233^	Arg^195^, (2) Glu^233^, His^299^, Asp^300^	(2) Arg^195^, Asp^197^, Glu^233^, Asp^300^	(2) Arg^267^, Thr^314^, Arg^346^	Thr^163^, Glu^233^	Gln^63^, His^305^, Gly^306^
Π-stacking	-	-	His^201^	-	-	Trp^59^
Salt bridges	-	-	-	Asp^317^	Asp^197^, Asp^300^	-
Interface area, Å^2^	167.7	152.4	234.5	149.6	167.6	206.1
Binding energy, kcal/mol	−5.8	−8.0	−8.2	−7.7	−8.8	−8.4
α-glucosidase–ligand complexes	Amino acids in contact with the ligand					
Hydrophobic interactions	Glu^216^	Met^363^, Val^588^, (2) Leu^865^, Val^867^, Leu^868^	Phe^90^, Phe^129^, (2) Val^236^	Met^363^, Arg^594^	Ile^98^, Tyr^110^, (2) Trp^126^, Trp^273^, Arg^275^, (2) Val^321^	Leu^865^
Hydrogen bonds	Ser^135^, (2) Thr^155^, Thr^156^, Glu^216^	Met^363^, His^584^	(2) Ser^88^	His^714^, Val^867^, Leu^868^	Ala^93^, Pro^94^, Arg^275^	Met^363^, (2) Arg^594^, Ser^864^, Glu^866^, Val^867^, Leu^868^, Glu^869^
Π-cation	-	-	-	His^717^		
Salt bridges	-	-	-	Asp^860^	Asp^91^	
Interface area, Å^2^	179.9	153.3	225.4	178.3	151.1	159.8
Binding energy, kcal/mol	−5.4	−7.2	−7.5	−6.7	−8.2	−7.7

## Data Availability

The original contributions presented in this study are included in the article. Further inquiries can be directed to the corresponding authors.

## Supplementary Materials

The following supporting information can be downloaded at: https://www.mdpi.com/article/10.3390/ph19040538/s1, Figure S1: The ^1^H-NMR and ^13^C-NMR spectrum of **p-TSA-a**.; Figure S2: The ^1^H-NMR and ^13^C-NMR spectrum of **p-TSA-b**; Figure S3: The ^1^H-NMR and ^13^C-NMR spectrum of **p-TSA-c**; Figure S4: The ^1^H-NMR and ^13^C-NMR spectrum of **p-TSA-c-d**; Figure S5: The ^1^H-NMR and ^13^C-NMR spectrum of **p-TSA-c-e**; Figure S6: Dose-response curves for the inhibition of α-amylase by the synthesized compounds and acarbose; Figure S7: Dose-response curves for the inhibition of α-glucosidase by the synthesized compounds and acarbose.
