# Supplementary material for: Combined In Vitro and Computational Investigations on Synthesized Sulfonamide-Based Antidiabetic Agents
Source: Pharmaceuticals (Basel). 2026 Mar 26;19(4):538. doi: 10.3390/ph19040538 (PMC13118603; doi:10.3390/ph19040538)
Supplement: Supplementary file 1 [file pharmaceuticals-19-00538-s001.zip › pharmaceuticals-4200267-supplementary.pdf]

Supplementary Materials

Results

^{1}H-NMR and ^{13}C-NMR Spectroscopy

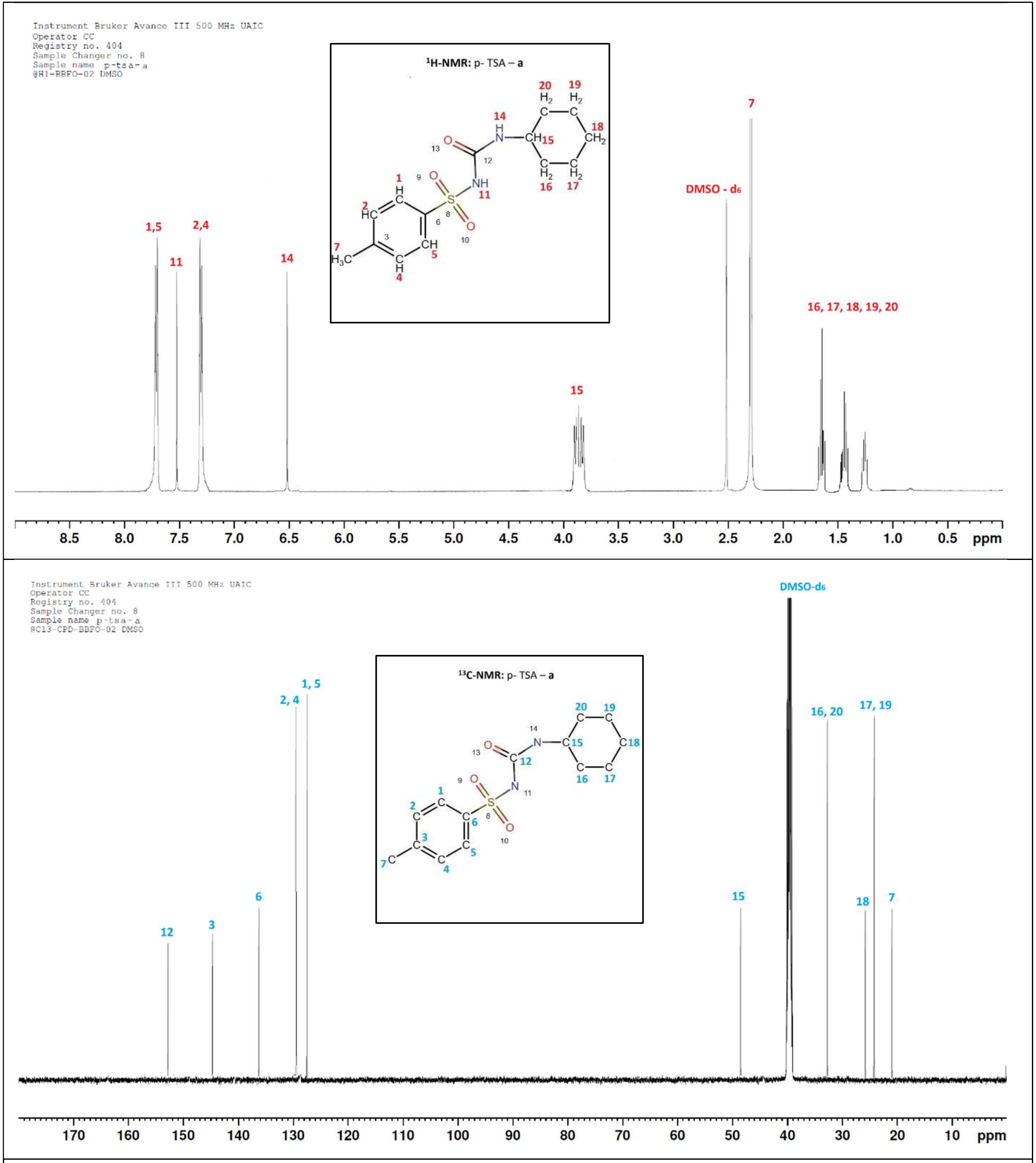

Figure S1. The ^{1}H-NMR and ^{13}C-NMR spectrum of p-TSA-**a.**

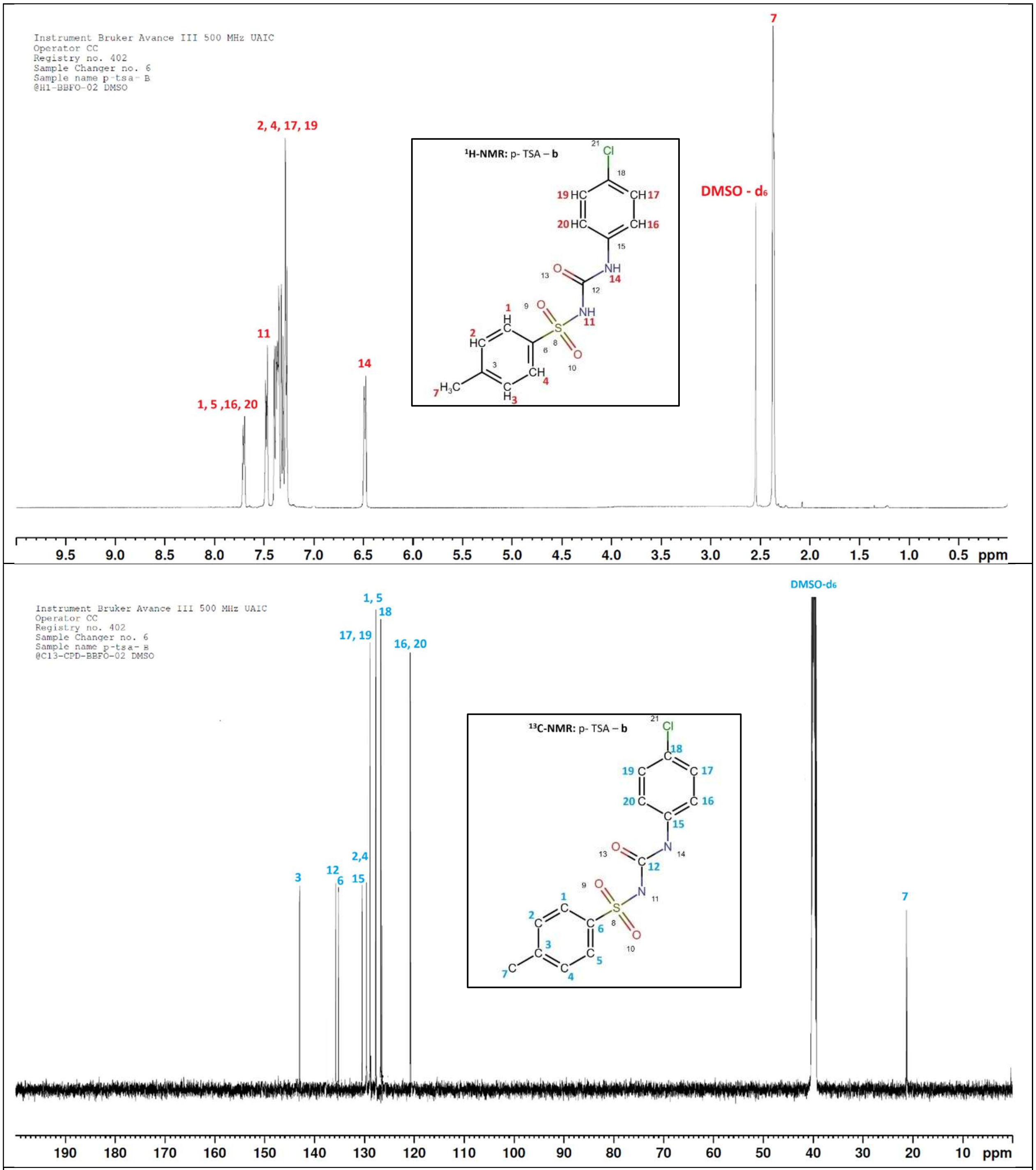

Figure S2. The ^{1}H-NMR and ^{13}C-NMR spectrum of p-TSA-**b.**

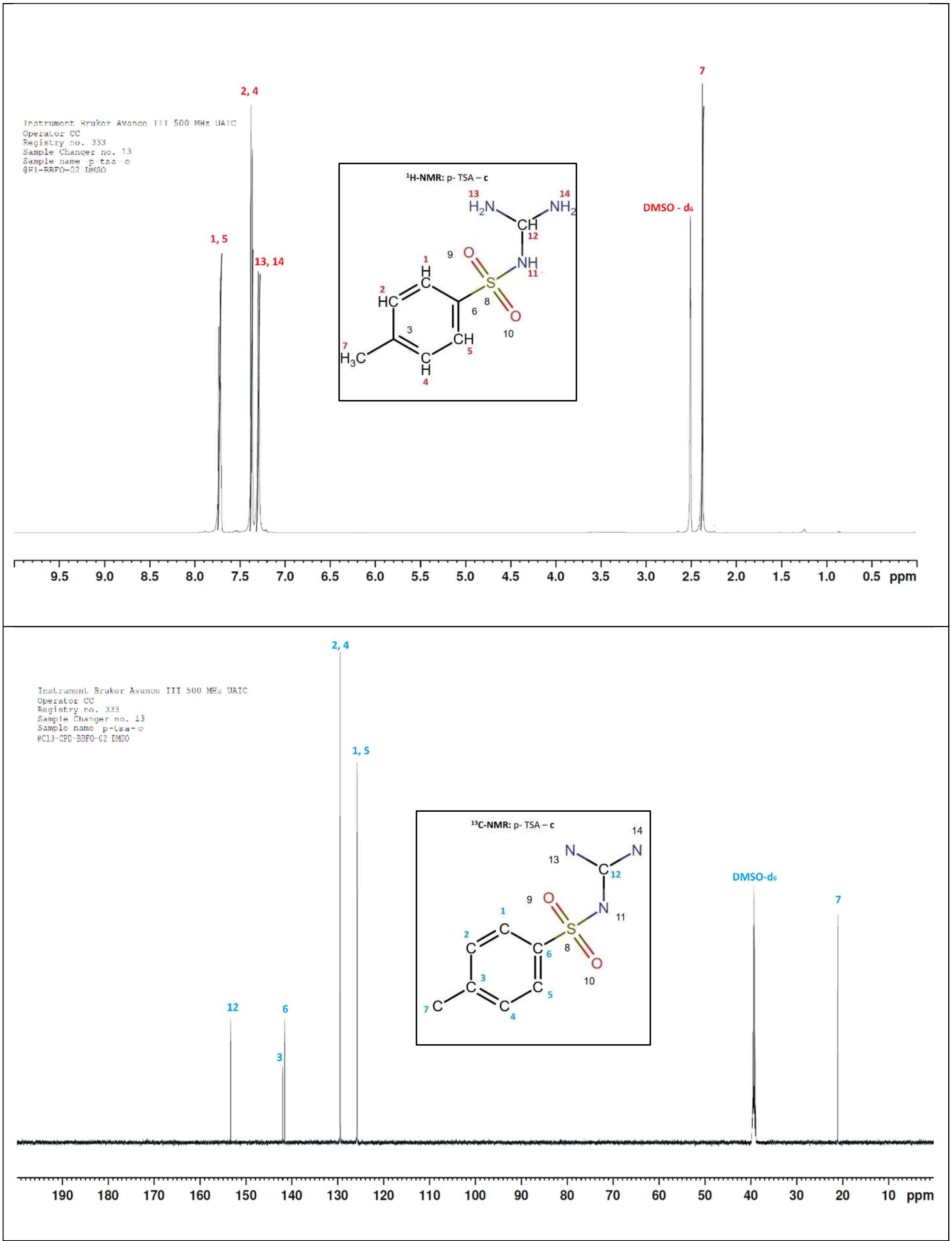

Figure S3. The ¹H-NMR and ¹³C-NMR spectrum of p-TSA-**c.**

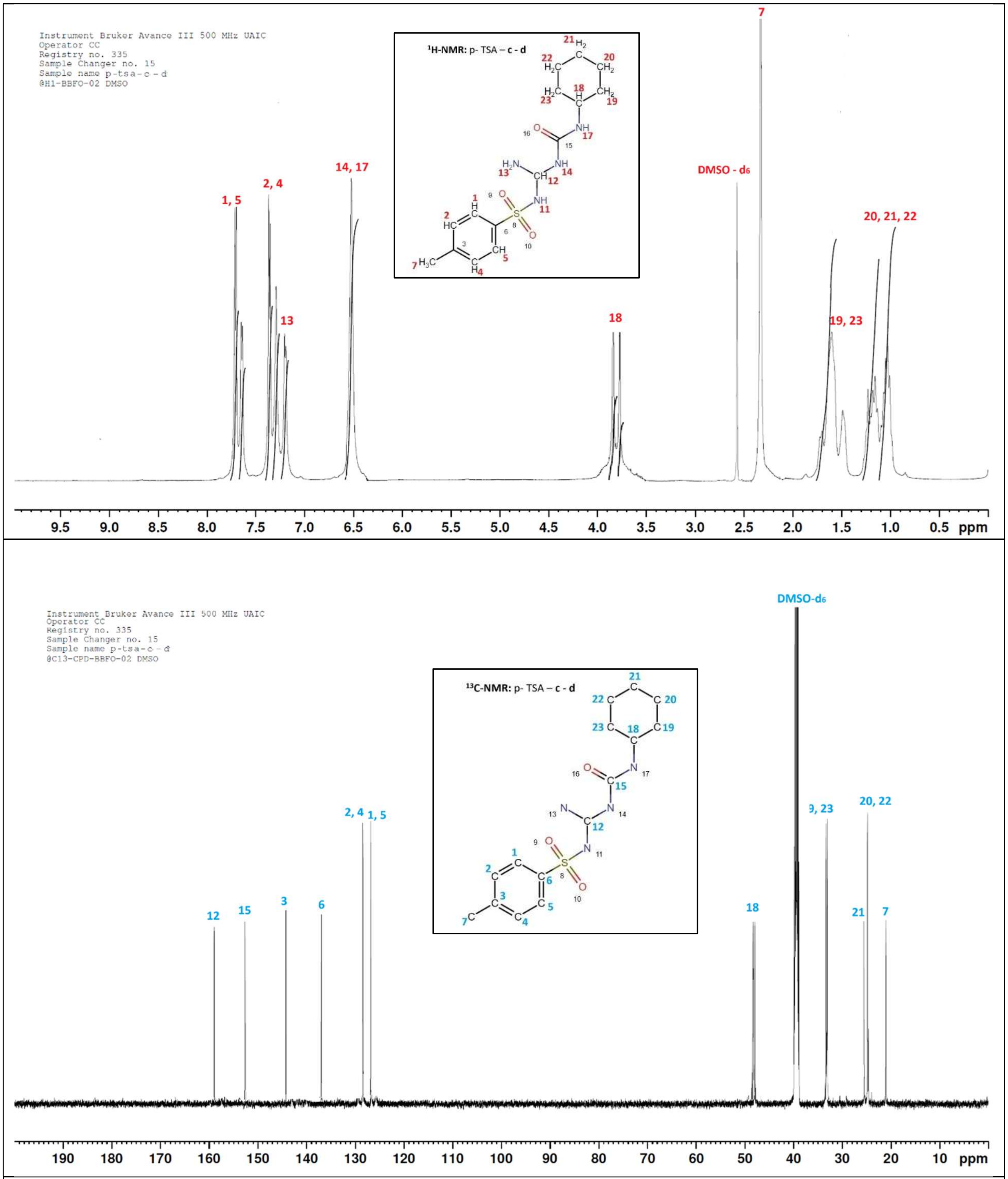

Figure S4. The ^{1}H-NMR and ^{13}C-NMR spectrum of p-TSA-**c-d.**

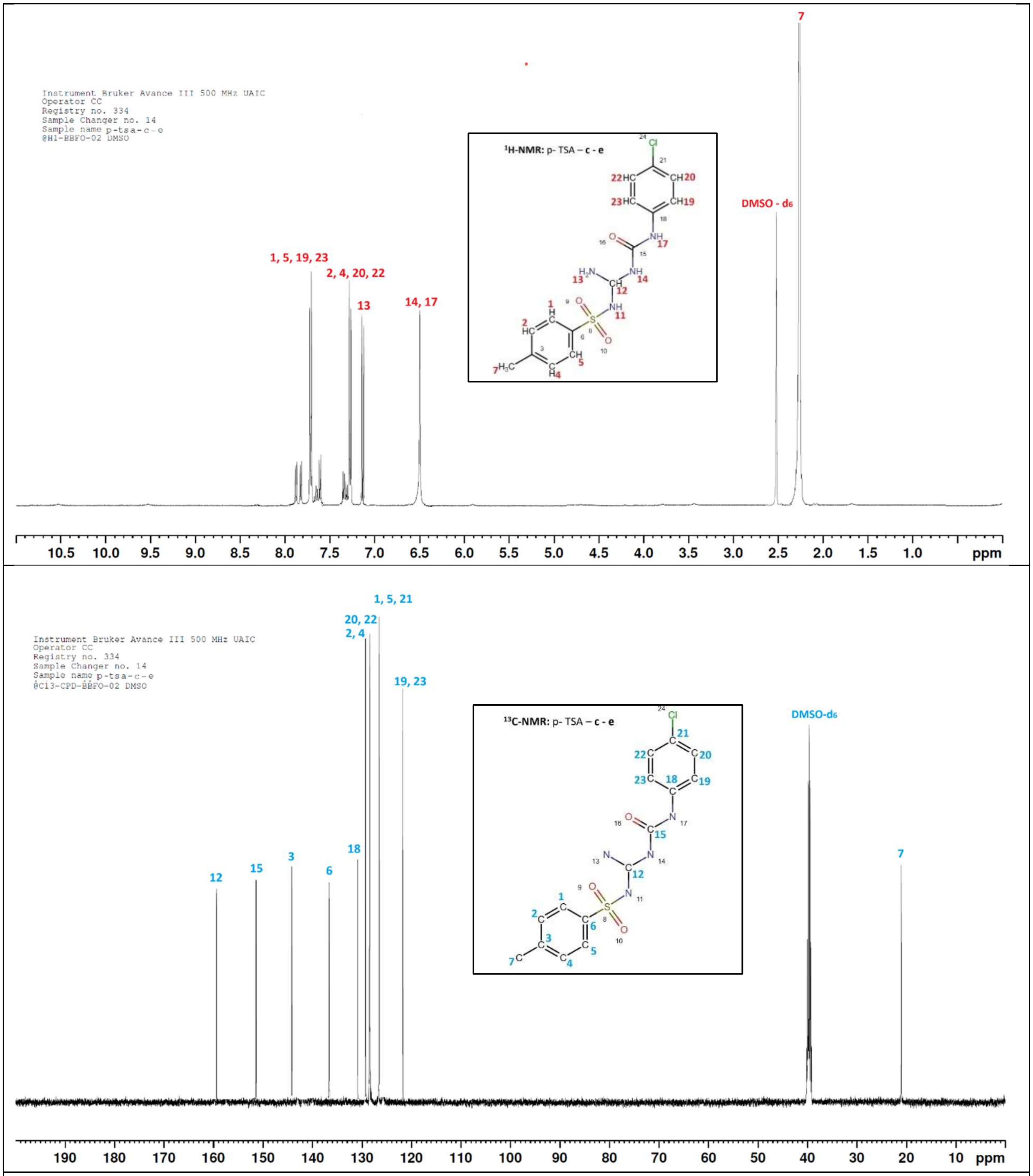

Figure S5. The ^{1}H-NMR and ^{13}C-NMR spectrum of p-TSA-**c-e**.

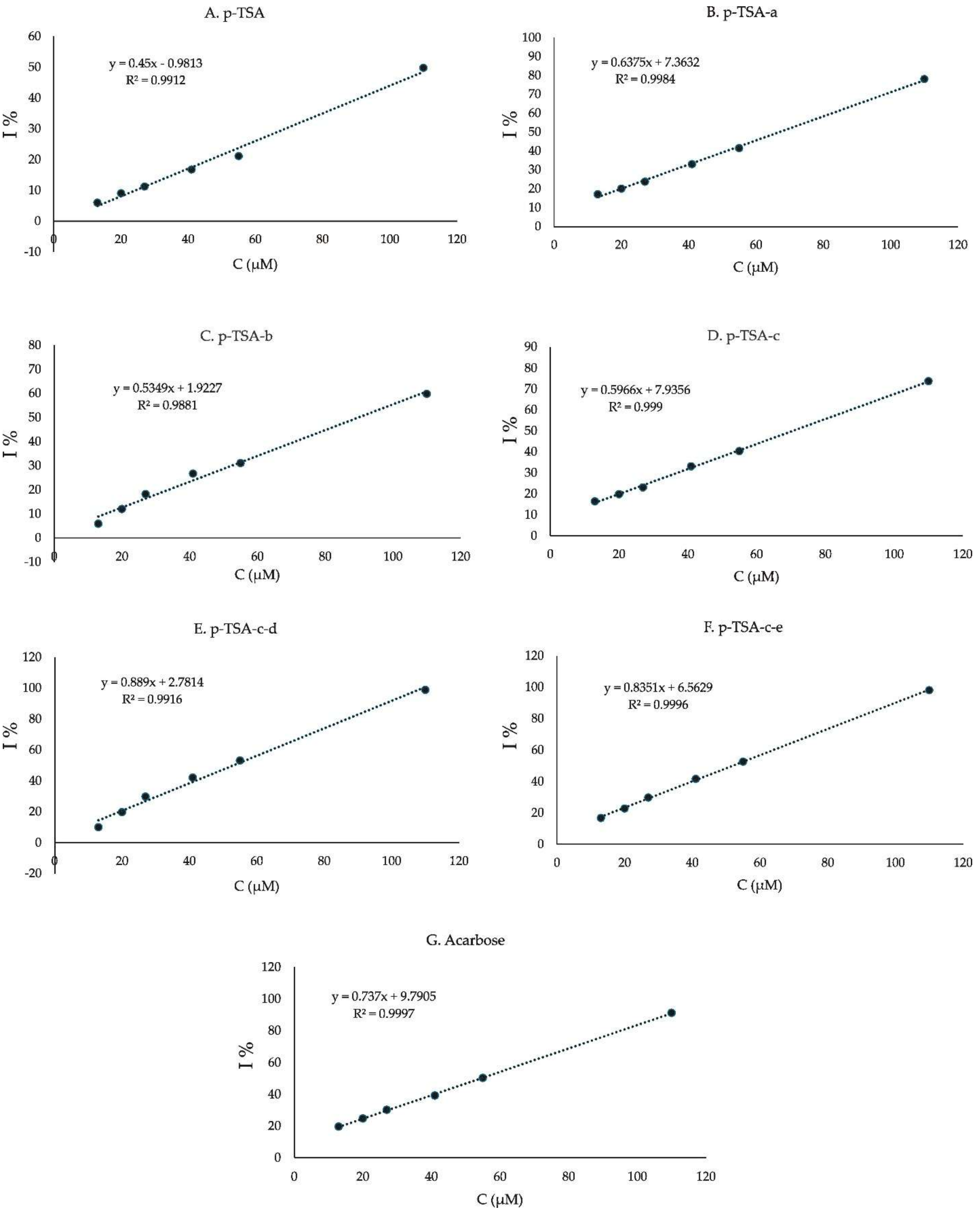

Figure S6. Dose-response curves for the inhibition of α-amylase by the synthesized compounds and acarbose.

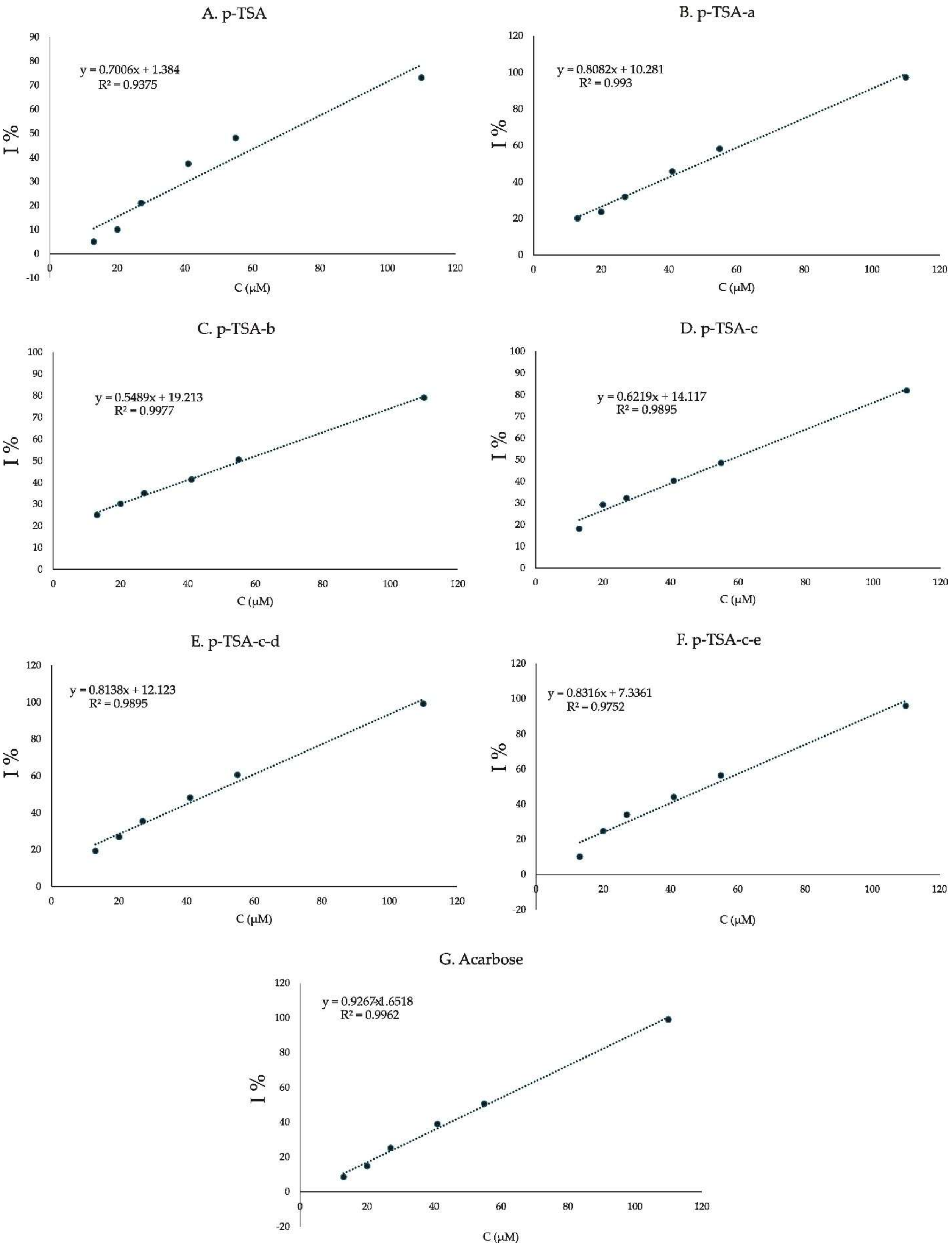

Figure S7. Dose-response curves for the inhibition of α-glucosidase by the synthesized compounds and acarbose.
